# Localization of Vasoactive Intestinal Polypeptide Receptor 1 (VPAC1) in Hypothalamic Neuroendocrine Oxytocin Neurons; A Potential Role in Circadian Prolactin Secretion

**DOI:** 10.3389/fnana.2020.579466

**Published:** 2020-10-29

**Authors:** Ida Stangerup, Jens Hannibal

**Affiliations:** Department of Clinical Biochemistry, Faculty of Health Sciences, Bispebjerg Hospital, University of Copenhagen, Copenhagen, Denmark

**Keywords:** prolactin, oxytocin, dopamine, releasing factor, paraventricular nucleus, VPAC1, circadian rhythm

## Abstract

Prolactin (PRL) is a versatile hormone and serves a broad variety of physiological functions besides lactation. The release of PRL from lactotrophs in the pituitary has in rodents been shown to be released with a circadian pattern depending on the physiological state of the animal. The circadian release of PRL seems to be complex involving tonic inhibition by dopamine (DA) neurons on lactotrophs and one or even several releasing factors. Because of the circadian releasing pattern of PRL, neurons in the suprachiasmatic nucleus (SCN), “the brain clock,” and especially the neurons expressing neuropeptide vasoactive intestinal polypeptide (VIP), have been suggested to be involved in the circadian regulation of PRL. In the present study, we used fluorescence immunohistochemistry, *in situ* hybridization histochemistry, confocal microscopy, three-dimensional reconstruction, and highly specific antibodies to visualize the occurrence of VIP receptors 1 and 2 (VPAC1 and VPAC2) in mouse brain hypothalamic sections stained in combination with VIP, oxytocin (OXT), arginine vasopressin (AVP), and DA (tyrosine hydroxylase, TH). We demonstrated that VIP fibers most likely originating from the ventral part of the SCN project to OXT neurons in the magnocellular part of the paraventricular nucleus (PVN). In the PVN, VIP fibers were found in close apposition to OXT neuron exclusively expressing the VPAC1 receptor. Furthermore, we demonstrate that neither OXT neurons nor TH or AVP neurons were expressing the VPAC2 receptor. VPAC1 receptor expression was also found on blood vessels but not in neurons expressing AVP or TH. These findings suggest that VIP signaling from the SCN does not directly target DA neurons involved in PRL secretion. Furthermore, the findings support the notion that VIP from neurons in the SCN could regulate circadian release of OXT in the posterior pituitary or modulate OXT neurons as a releasing factor involved in the circadian regulation of PRL from pituitary lactotrophs.

## Introduction

Prolactin (PRL) is an extremely versatile hormone with regulatory functions in the reproductive system, immune system, and during lactation (Freeman et al., 2000). It is primarily secreted from lactotrophs in the anterior lobe of the pituitary gland, and its key role in lactation gave name to the hormone (Freeman et al., 2000; Grattan, 2015). The role of PRL in lactation as well as the inhibitory role of dopamine (DA) on pituitary PRL release has been well defined for years (Fitzgerald and Dinan, 2008; Grattan, 2015). PRL is markedly different from the other pituitary hormones because it lacks an endocrine target tissue and a classical neuroendocrine feedback loop. Furthermore, no single releasing factor has yet been identified, although many neuropeptides and classical neurotransmitters can influence the secretion of PRL (Freeman et al., 2000). Thus, many unanswered questions remain regarding the regulatory pathway of PRL secretion. PRL is secreted upon the stimulation of the neuroendocrine reflex generated by suckling, and in addition, rat studies have shown that PRL is secreted with different rhythmic pattern (Freeman et al., 2000; Egli et al., 2004; Bertram et al., 2010). Two rhythms of PRL secretion have been defined which are different from each other depending on the physiological state of the rat (Freeman et al., 2000; Egli et al., 2004; Bertram et al., 2010). In female rats, one rhythm generated by the ovarian cycle results in an afternoon proestrus PRL surge every fourth to fifth day. Both mating and artificial cervical stimulation have been shown to generate another PRL secretion rhythm—a circadian rhythm—with a nocturnal PRL surge in the morning and a diurnal PRL surge in the afternoon (Freeman et al., 2000; Egli et al., 2004; Bertram et al., 2010). In male rats, PRL secretion is primarily circadian regulated with a peak in serum concentration at the transition zone between day and night (Esquifino et al., 2004).

Circadian regulated hormonal secretion in mammals is driven by the hypothalamic suprachiasmatic nucleus (SCN), also known as the “brain’s clock.” Solid evidence has demonstrated that signals from the SCN are important for the circadian regulation of PRL secretion and disappear in SCN lesioned animals (Freeman et al., 2000). Vasoactive intestinal polypeptide (VIP) found in neurons of the ventral SCN has been shown to be involved in the circadian regulation of PRL *via* a pathway that involves oxytocin (OXT; Egli et al., 2004). VIP is a member of the secretin/glucagon/pituitary adenylate cyclase activating polypeptide (PACAP) family and shares three common G-protein-coupled receptors (VPAC1, VPAC2, and PAC1) with its sister peptide Pituitary adenylate cyclase activating polypeptide (Dickson and Finlayson, 2009). VIP binds to VPAC1 and VPAC2, whereas Pituitary adenylate cyclase activating polypeptide primarily binds to the PAC1 receptor (Hannibal and Fahrenkrug, 2003). VIP fibers from the SCN project to the paraventricular nucleus (PVN), the origin of neurosecretory OXT neurons (Buijs et al., 1999). OXT has been suggested to be a potential circadian releasing factor for PRL released from lactotrophs in the anterior pituitary (Arey and Freeman, 1989, 1992a; Egli et al., 2004). In previous studies, VIP was shown to be involved in OXT-mediated release of PRL, although the mechanism and receptor involved are not clarified (Arey and Freeman, 1989, 1990, 1992b; Egli et al., 2004).

In the present study, we analyze the occurrence of VPAC1 and VPAC2 receptors using well-characterized antibodies against the VPAC1 and the VPAC2 receptors and examine the localization and expression of these receptors in the PVN, SON, and median eminence (ME). Furthermore, we show that VIP fibers most likely originating from SCN target OXT neurons exclusively expressing the VPAC1 receptor.

## Materials and Methods

### Animals

Mice (mixed C57BL/6J 129/SV, five males and five females) were housed under standard laboratory conditions in a 12-h light–dark circle with water and food *ad libitum* (Altromin 1324; Altromin Specialfutter, Germany). Staining of VPAC1 (see below) was not different when comparing genders, and the females were examined independent of the ovarian cycle. Animals were treated according to the principles of Laboratory Animal Care (Law on Animal Experiments in Denmark, LBK NR474, May 15, 2014) and Dyreforsoegstilsynet, Ministry of Justice, Denmark, who issued the license number 2017/15-0201-01364 to Jens Hannibal thereby approving the study. Mice were sacrificed midday (Zeitgeber time = ZT4–8, ZT = 0; lights ON, ZT12; lights OFF) and were minimum 10 weeks old when included in the study. For *in situ* hybridization histochemistry three mice (mixed C57BL/6J 129/SV) were decapitated at midday (ZT6), and the brains were rapidly removed and frozen at −80°C before cutting in a cryostat.

### Antibodies

The antibodies against VIP, VPAC1, and the VPAC2 receptor were raised in rabbits immunized with VIP and with recombinant fusions proteins of the N-terminal of the VPAC receptors expressed in *Escherichia coli*. Specific details on generation and purification of the receptor antibodies are previously described (Fahrenkrug et al., 1995, 2000; Hannibal et al., 2017). For further test of specificity, we also stained VPAC1 knockout mice obtained from Jim A. Waschek, UCLA. The VPAC1 mice were originally characterized by Fabricius et al. (2011). These mice had exon 4–6 deletion making a functional knockout, but a truncated part of the N-terminal receptor protein was still expressed (Fabricius et al., 2011), resulting in a positive staining using our VPAC1 antibody (Supplementary Figure 1). For further characterization, we therefore demonstrate the localization of VPAC1 mRNA in the PVN/SON (see below). Commercial antibodies against OXT, arginine vasopressin (AVP), and tyrosine hydroxylase (TH) were raised in guinea pigs (OXT, AVP) and in rabbits (TH; Table 1).

**Table 1:** Primary antibodies.

Antibody	Host	Source	Catalog/code no.	Dilution (K)	Incubation (days)
VIP	Rabbit	Inhouse RRID:AB_2313759	291E	1:2	2
VPAC1	Rabbit	Inhouse RRID:AB_2814929	4G28	1:2/1:10	4
VPAC2	Rabbit	Inhouse RRID:AB_2814676	623S	1:20	4
Oxytocin	Guinea pig	Peninsula Laboratories International	GHC8152	1:2	2
Vasopressin	Guinea pig	Peninsula Laboratories International	T-5048	1:1	2
Tyrosine hydroxylase	Rabbit	Chemicon	AB 151	1:1	2

### Immunohistochemistry

Before fixation, animals were anesthetized using a subcutaneous injection of hypnorm and midazolam, 0.1 ml per 10 g of body weight. Hereafter the animals were transcardially perfused with heparin [15,000 IE/I phosphate-buffered saline (PBS), pH 7.2] for 3 min followed by Stefanini fixative [2% paraformaldehyde (PFA), 15% picric acid in 0.1 M PBS, pH 7.2] for 15 min. All brains were postfixed overnight in Stefanini fixative and cryoprotected in 30% sucrose prior to freezing (−80°C). Series of coronary sections (40-μm-thickness) used for immunohistochemical staining were cut on a Leica CM3050S cryostat (−18°C). Before processing for immunohistochemistry, brain sections were treated by antigen retrieval solution for 1.5 h 80°C (DAKO ChemMate, Glostrup, Denmark, code no. S 203120 in distilled water, pH 6). For visualization of either VPAC1 or VPAC2 and VIP or VPAC1 and TH, we used the method previously described on two primary antibodies raised in the same species using biotinylated tyramide amplification system and Envision (Dako, ChemMate, Glostrup, Denmark) and streptavidin-conjugated AlexaFluor 488 (016-540-084, Jackson ImmunoResearch, Baltimore, MD, USA) and secondary anti-rabbit antibody-conjugated to Alexa594 (A21207, Thermo Fisher Scientific, Rockford, IL, USA; Hannibal et al., 2017).

Sections were incubated with primary antibodies for 2–4 days at 4°C followed by incubation with secondary antibodies overnight. Details on primary and secondary antibodies can be found in Tables s 1, 2. As control, sections were routinely stained after omitting the primary antibody, which eliminated all staining.

**Table 2:** Secondary antibodies.

Antibody	Source	Species	Dilution
Biotinylated donkey antiserum	Jackson ImmunoResearch Laboratories Cat#711-065-152 RRID:AB2340593	Rabbit	1:1,500
Envision	Dako (K4002)	Rabbit	1:2
Alexa Fluor 594–conjugated donkey IgG	Thermo Fisher Scientific Cat#A21207 RRID:AB_10049744	Rabbit	1:800
Alexa Fluor 594–conjugated donkey IgG	Jackson ImmunoResearch Laboratories Cat#706-585-148 RRID:AB_2340474	Guinea pig	1:200

Photomicroscopy was performed using an iMIC confocal microscope (Till Photonic, FEI Munich, Germany) equipped with the following objectives: ×10, numerical aperture (NA) = 0.35; ×20, NA = 0.75; ×40, NA = 1.3; and ×60, NA = 1.46. Using the ×60, the highest resolution [(*r* = λ/NA), where λ is the imaging wavelength] was for ×60 = 174 nm. Resolution in the *z* axis was at ×60 0.2 μm. All images in *Z*-stacks photographed using the ×40 or ×60 objective were deconvoluted in AutoQuant X, version 3.04 (Media Cybernetics, Inc., Rockville, MD, USA) before analysis in IMARIS^®^ version 9.1.2 (RRID:SCR_007370) from Bitplane, Switzerland^1^. Colocalization and analysis of close apposition (synaptic appositions) and *Z-stacks* in three-dimensional (3D) reconstructions obtained by ×60 objective were analyzed in the colocalization module of IMARIS^®^. Colocalization was established if two colors representing the different antigens were found in the same pixel. In ×60, one pixel had a dimension of 100 × 100 nm. In cases where VIPs were colocalized with VPAC1 in anatomical structures compatible with the localization of synapses, the colocalization most likely represents genuine synaptic appositions. All images were adjusted for brightness and contrast either in Fiji or in Photoshop CS5 (Adobe, San Jose, CA, USA, RRID:SCR_014199) and mounted into plates in Adobe Illustrator CS5 (Adobe).

### Cell Counting

Coronal sections representing the rostrocaudal PVN (and SON) were used for evaluation and cell counting from three males and two females. In case of colocalization of antigens in the same cell, manual evaluation was performed using the cell counting module of Fiji software (version 1.47q, NIH, USA, RRID:SCR_003070). Each of two 8-bit images representing the two different antigens (i.e., VPAC1 and OXT) were counted using the cell counter modules where after the cells costoring both markers and thus representing cells expressing both VPAC1 and OXT were counted and colocalization calculated (sections from the PVN/SON from one male and one female were used; Hannibal et al., 2019).

### Fluorescence *in situ* Hybridization Using Digoxigenin-Labeled Antisense RNA Probes

For detection of mouse *VPAC1* mRNA antisense, RNA probes were used. As template, nucleotide 42–1509 (AK052465) excised as an *Eco*RI fragment (Source Bioscience, Nottingham, UK) and inserted in the *Sma*I site of pBluescriptKS+ was used. The resulting plasmid was linearized with *Xba*l for antisense and with *Hind*III for the sense probes, and transcription was done using T3 and T7 polymerase, respectively. *In situ* hybridization for *VPAC1* mRNA was performed using digoxigenin-labeled probes (Tsai et al., 2009). Briefly, brain sections were cut on a cryostat in 12-μm-thick coronal sections through the rostral to the caudal part of the PVN. From each animal, one gelatin-coated slide from each series representing the rostral, mid, and caudal part of the PVN and SON, respectively, was hybridized with the Dig-labeled *VPAC1* antisense probe (diluted 1:1,000). After 15-min fixation in 4% PFA, hybridization overnight, and washing, slides were incubated with a sheep anti-digoxigenin antibody (Roche, code: 11 214 667 001; diluted 1:2,000) followed by washing and incubation with a biotinylated donkey anti–sheep antibody (Jackson ImmunoResearch code; 713-066-147, diluted 1:800), washed followed by incubation in ABC reagent (Vectastain PK6100) biotinylated tyramide (Perkin Elmer SAT 700, diluted 1:50) and finally in streptavidin conjugated Alexa Fluor 488 (Jackson ImmunoResearch; code 016-540-084, diluted 1:500). After washing, slides were mounted in glycerol DAPI solution (Hannibal et al., 2019).

## Results

### Expression of VPAC1 but Not VPAC2 Receptors in the PVN and SON

**Figure 1:**
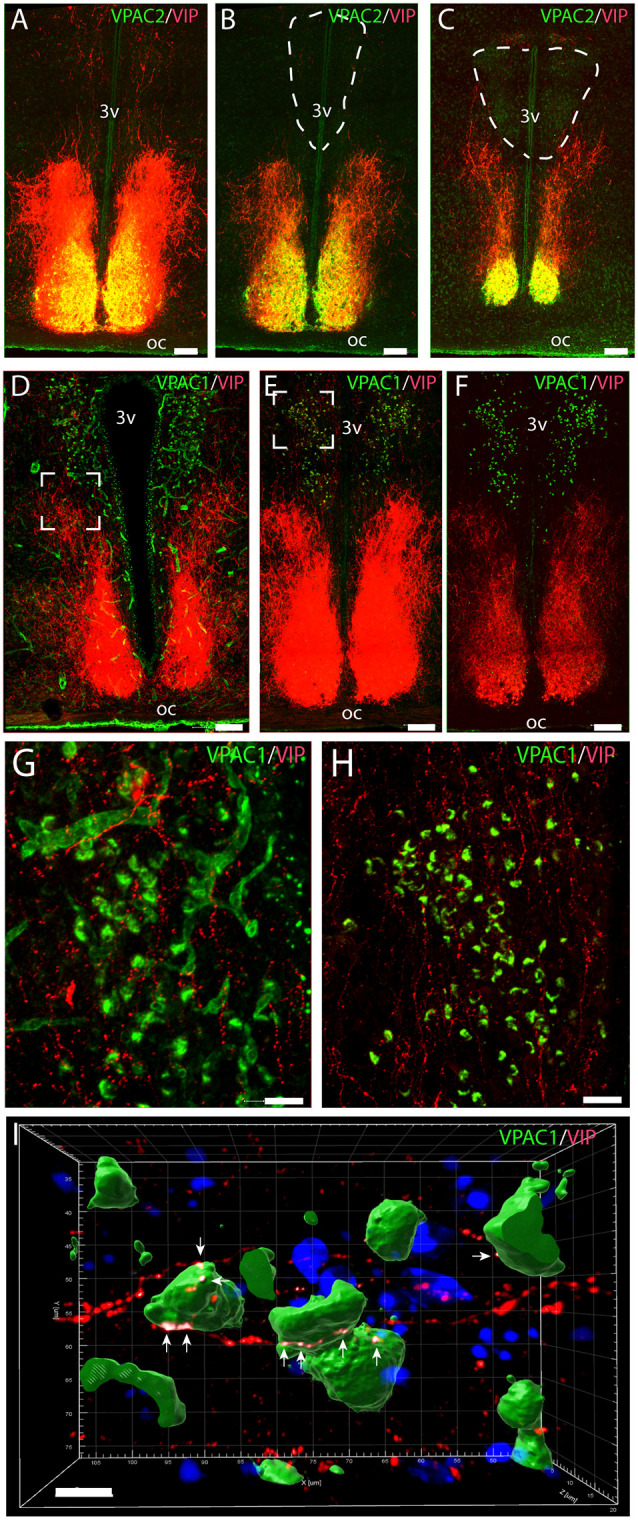
Distribution of VPAC2 (green) and vasoactive intestinal polypeptide (VIP; red) immunoreactivity **(A–C)** and distribution of VPAC1 (green) and VIP (red) immunoreactivity **(D–F)** in the mid and rostral part of the suprachiasmatic nucleus (SCN) and paraventricular nucleus (PVN) in a wild-type mouse. Images **(A–E,G,H)** represent maximal projections of 14 digital sections in a *Z-stack* (*Z* = 2 μm). Image I represents a 3D reconstruction of a *Z-stack* (*Z* = 0.2 μm) of 100 images. Strong VIP and VPAC2 immunoreactivity were found in SCN **(A)** with VIP fibers projecting toward both the rostral part of PVN **(B)** and mid part of PVN **(C)**. VPAC1 immunoreactivity was found in both the rostral **(E)** and mid **(F)** part of PVN using ENVISION for visualization. Colocalization module (see “Materials and Methods” section) was used to determine close apposition between VIP fibers and VPAC1. Frames **(G,H)** represent the white frames in **(D,E)**, respectively. Because of amplification with biotinylated tyramide, VPAC1 in the blood vessels becomes visible **(G)**. 3D reconstruction of frame **(H)** represented in frame I shows VIP fibers making synaptic appositions (white dots indicated by arrows) on several VPAC1 receptor expressing neurons in PVN. Determination of possible synaptic appositions between VIP and VPAC1 was made using the IMARIS colocalization module (see “Materials and Methods” for details). Scale bars: **(A–E)** = 100 μm, **(G–H)** = 25 μm, **(I)** = 7 μm.

**Figure 2:**
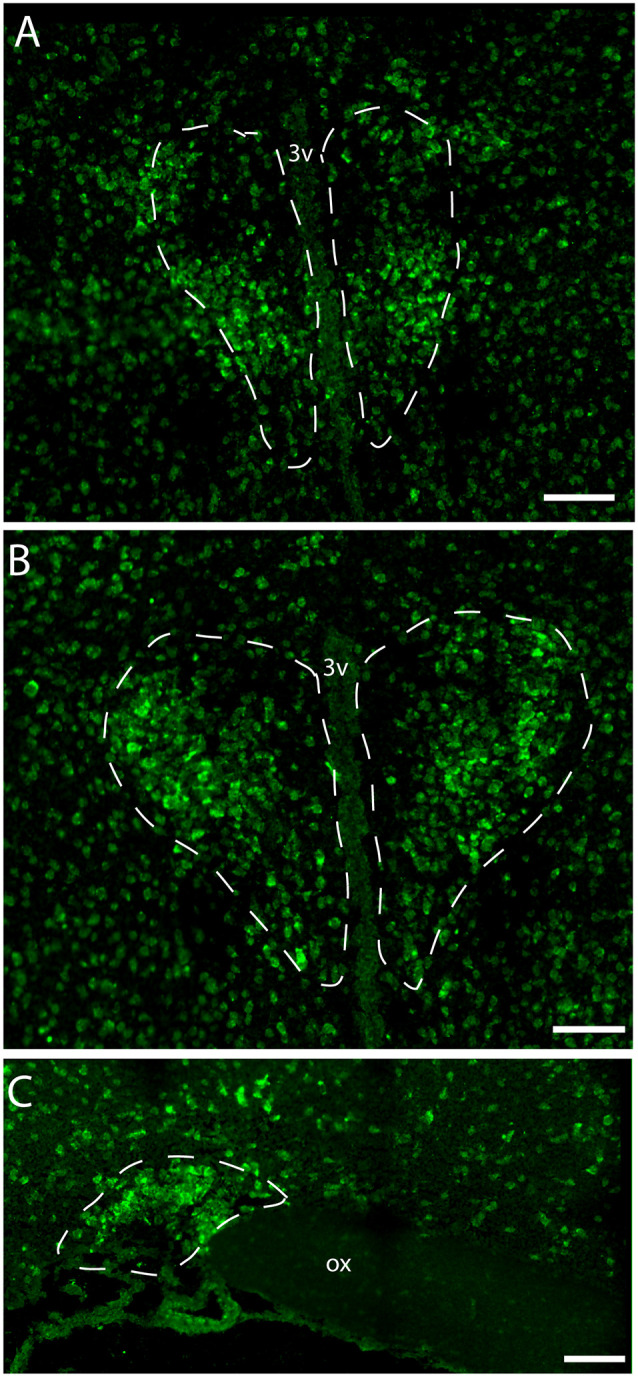
Distribution of VPAC1 mRNA in the rostral and mid PVN **(A,B)** and SON **(C)** using a digoxiginin-labeled cRNA probe. Dashed lines indicate in **(A,B)** the rostral and mid PVN, respectively, and **(C)** the SON. Ox. optic chiasm; 3v, third ventricle. Scale bars: **(A–C)** = 100 μm.

**Figure 3:**
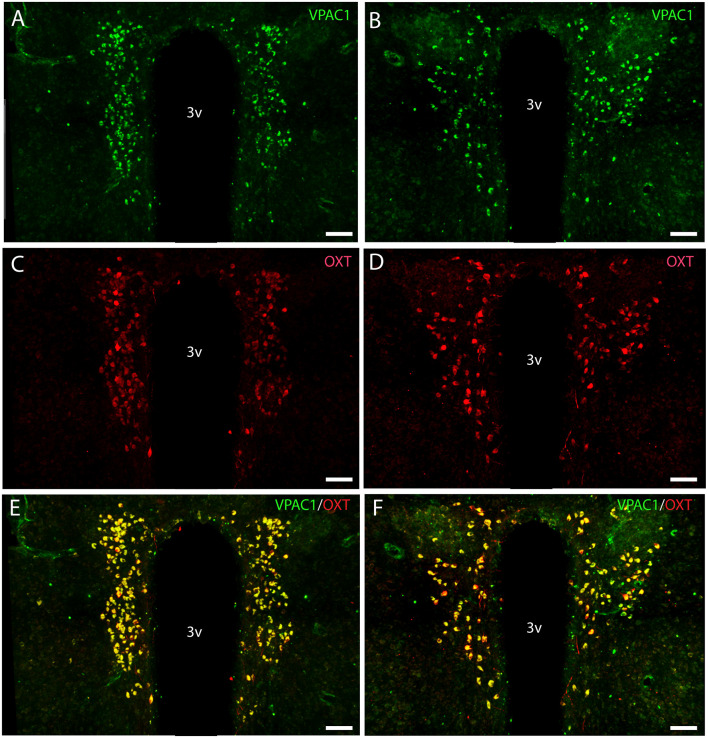
Distribution of VPAC1 (green) and oxytocin (OXT; red) immunoreactivity in the mid and rostral part of SCN and PVN in a wild-type mouse. Images **(A–F)** represent maximal projections of 16 digital sections in a *Z*-stack (*Z* = 2 μm). **(A,B)** show immunoreactivity for VPAC1 in the rostral and mid part of PVN. **(C,D)** show immunoreactivity for OXT in the rostral and mid part of PVN. Colocalization between VPAC1 and OXT in the rostral and mid part of PVN is visualized in **(E,F)** using IMARIS colocalization module (see “Materials and Methods” section). Scale bars: **(A–F)** = 80 μm.

**Figure 4:**
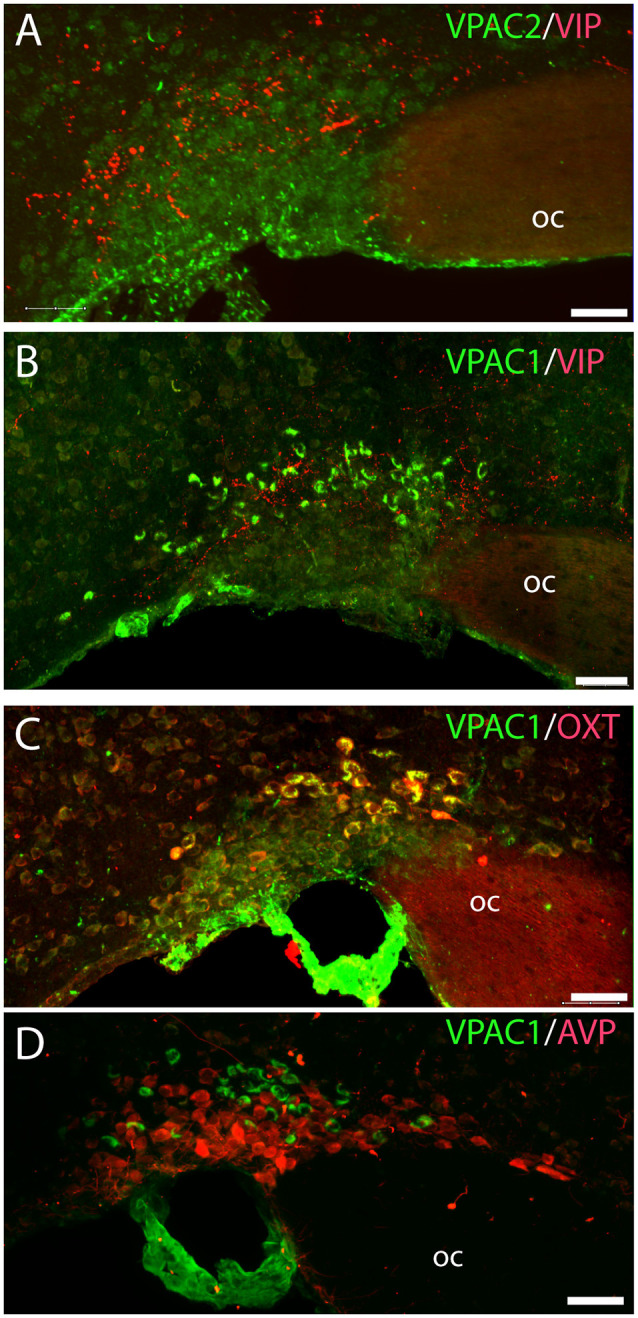
Distribution of VIP (red), VPAC1 (green), VPAC2 (green), OXT (red), and arginine vasopressin (AVP; red) immunoreactivity in the SON. VPAC2 (green) was not visualized in the SON **(A)**. VIP and VPAC1 immunoreactivity were found in the SON **(B)**. VPAC1 (green) was also found in the cerebral blood vessel surrounding the SON **(C,D)**. A *Z*-stack of high-resolution images used for 3D reconstruction showed colocalization of VPAC1 (green) and OXT (red) visualized as yellow in **(C)**. AVP and VPAC1 immunoreactivity was found in the SON; 3D reconstruction showed them to be localized in separate neurons. Images **(A–D)** represent maximal projections of 16 digital sections in a *Z-stack* (*Z* = 2 μm). Scale bars: **(A–D)** = 25 μm.

Immunostaining of VPAC1 in the mouse hypothalamus reveals a different pattern of staining, depending on the methods used for visualization. Using amplification by biotinylated tyramide (Berghorn et al., 1994; Hannibal et al., 2014) VPAC1 immunoreactivity (VPAC1-IR) was found in blood vessels of the basal arteries as described previously (Fahrenkrug et al., 2000), as well as in blood vessels of the entire brain including the hypothalamus (Figures 1D,G) and in neurons of the PVN and SON (Figures 1E,F, 4B). Using the ENVISION-based amplification system, which is less powerful, VPAC1-IR was found weakly stained in blood vessels compared with the tyramide system, whereas the neurons of the PVN and SON were more clearly presented (Figures 1E,F,H, 4B). Because the observations in neurons were the focus in this study, the remaining examinations were performed using the ENVISION detection system. Within the PVN, VPAC1 expression was found in neurons located in the rostral to the caudal magnocellular part of the PVN, very similar to the localization of OXT-expressing neurons (Figures 3, 4C). In the SON, VPAC1-expressing neurons were found in the dorsal part of the nucleus (Figures 4B,C). In both the PVN and SON, delicate VIP-expressing nerve fibers were found in close apposition to many VPAC1-expressing neurons and blood vessels (Figures 1G–I, 4B). VIP-expressing nerve fibers in the PVN seemed to originate directly from VIP neurons in the SCN and to have synaptic appositions with VPAC1-expressing neurons (Figures 1G–I). To verify the localization of VPAC1 expression in the PVN and SON, *in situ* hybridization histochemistry using Dig-labeled antisense VPAC1 receptor RNA probes was used. The localization of VPAC1 mRNA expression in the PVN and SON (Figures 2A–C) was found to be similar to that found using immunohistochemistry for the VPAC1 receptor protein (Figures 3, 4B) in both the rostrocaudal extent of the PVN and in the SON.

VPAC2 immunoreactivity was found exclusively on the soma and dendritic processes of the neurons in the SCN as described previously (Hannibal et al., 2017; Figures 1A–C). No VPAC2 immunoreactivity was found in either the PVN (Figures 1A–C) or SON (Figure 4A).

### VPAC1 Is Exclusively Expressed on OXT Neurons in the PVN and SON

**Figure 5:**
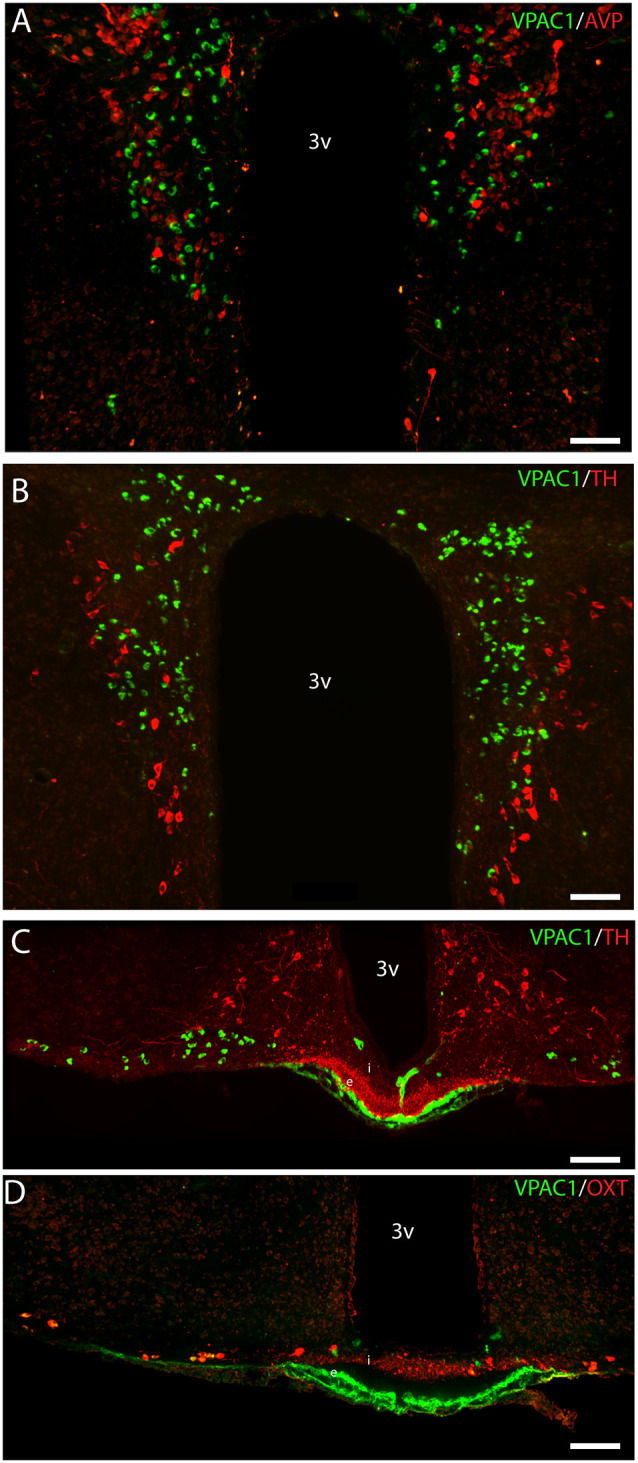
Distribution of VPAC1 (green), tyrosine hydroxylase (TH; red), OXT (red), and AVP (red) in the PVN and median eminence (ME). VPAC1 (green), TH (red), and AVP (red) immunoreactivity were found in the PVN **(A,B)** showing no colocalization. TH (red) was found in the external layer of the ME **(C)**, whereas OXT (red) was found in the internal layer **(D)**. VPAC1 (green) immunoreactivity was seen in relations to the portal blood vessel **(C,D)**, as an indicator of VPAC1 expression on the smooth muscle cells of the cerebral blood vessels but was not colocalized with TH or OXT neurons in the ME. Images **(A–D)** represent maximal projections of 16 digital sections in a *Z*-stack (*Z* = 2 μm). Scale bars: **(A,B)** = 80 μm; **(C,D)** = 100 μm.

OXT neurons were present throughout the magnocellular part of the mouse PVN (Figures 3C,D) with a higher density of cells in the rostral part of PVN (Figure 3C) compared to the mid part of PVN (Figure 3D). Reconstruction of a stack of high-resolution images confirmed VPAC1–IR in all OXT neurons in the entire PVN (Figures 3E,F) and in the SON (Figure 4C). In the rostral PVN, a total of 211 neurons were counted, and 100% colocalization of both VPAC1 and OXT was found. In the mid-caudal PVN, a total of 119 neurons expressed VPAC1-IR and 127 neurons expressed OXT. One hundred percent of the VPAC1 cells contained OTX, and 94% of all OTX cells contained VPAC1-IR. In the SON, a total of 31 neurons costored VPAC1 and OXT (100%), and one neuron (96%) contained only OXT. AVP neurons were found in a separate population of magnocellular neurons of the mouse PVN and SON as described by others (Godefroy et al., 2017; Figures 4D, 5A). No VPAC1-IR was found in AVP neurons in either the PVN or the SON (Figures 4D, 5A). Neurons synthesizing TH, representing DA neurons, were present in the PVN and the periventricular nucleus but did not show any immunoreactivity of the VPAC1 receptor (Figure 5B).

### VPAC1 Expression in the Median Eminence

VPAC1-IR was also found in the ME located in few neurons of the basal hypothalamus (Figure 5C). TH neurons most likely representing tuberoinfundibular neurons (TIDA neurons; Grattan, 2015) were found in the basal hypothalamus/arcuate nucleus (Figure 5C) and in nerve fibers innervating the portal blood vessels in the external layer of the ME (Figure 5C). VPAC1-IR was found neither in neurons nor nerve terminals expressing TH (Figure 5C). Few OXT neurons were found in the basal hypothalamus, and all expressed VPAC-IR (Figure 5D). OXT was also found in nerve terminals of the internal layer of the ME, but these nerve fibers did not contain the VPAC1 receptor (Figure 5D).

## Discussion

This study shows that VIP-containing nerve fibers most likely originating from the SCN target OXT neurons exclusively expressing the VPAC1 receptor. Neither the VPAC1 nor the VPAC2 receptor was found on DA neurons in the periventricular, paraventricular or in the arcuate nucleus. Our observations indicate that VIP signaling from the SCN does not directly regulate DA neurons involved in PRL secretion. These findings could be a “missing link” in the signaling pathway involved in circadian regulation of PRL from pituitary lactotrophs *via* VIPergic neurons located in the SCN projecting to magnocellular OXT-expressing neurons in the PVN (Arey and Freeman, 1989, 1990, 1992b; Egli et al., 2004).

DA neurons located in the arcuate nucleus are the predominant regulators of PRL secretion through a tonic inhibition on lactotrophs (Freeman et al., 2000; Fitzgerald and Dinan, 2008; Grattan, 2015). Three different subpopulations of DA neurons based on their localization within the arcuate nucleus—the TIDA, the tuberophyseal, and the periventricular (PHDA)—have been defined (Freeman et al., 2000; Grattan, 2015). Decline of DA in the portal blood occurs simultaneously with a circadian surge of PRL at the transition between day and night (De Greef and Neill, 1979; Esquifino et al., 2004). Because of this classic circadian secretion pattern, it has been suggested that VIP originating from the SCN could be a potential coregulator by inhibiting the tonic inhibition of DA neurons on lactotrophs (Gerhold et al., 2001, 2002). This hypothesis is based on anatomical observations in rats showing that VIP nerve fibers from VIP-expressing neurons located in the SCN were found in close contact with DA neurons in the arcuate nucleus (Gerhold et al., 2001). Furthermore, TH neurons were shown to express the VPAC2 but not the VPAC1 receptor (De Greef and Neill, 1979). In mice, we observed TH immunohistochemical staining representing DA neurons in PHDA neurons and VIP fibers from SCN passing by to the PVN, but neither VPAC1 nor VPAC2 was coexpressed in any TH immunoreactive neurons. Species differences between rats and mice could be a potential explanation, although antibody specificity of the former study (De Greef and Neill, 1979) should be considered. We used highly specific anti-VPAC1 and -VPAC2 receptor antibodies. We confirm the specificity of our IHC localization of VPAC1 in the PVN and SON by demonstrating VPAC1 mRNA by *in situ* hybridization in the same compartments of the PVN and SON. We have previously demonstrated that VPAC2 is present in the SCN (Hannibal et al., 2017), and in this study, we demonstrate that VPAC2 is not present in the PVN or SON. This is in agreement with another study (An et al., 2012) and, furthermore, the expression of VPAC2 mRNA as reported in Allen’s Brain Atlas^2^. VPAC2 mRNA and receptor protein (own observations) are, despite being present in SCN, also found in the bed nucleus of the stria terminalis and the amygdala complex but not in the periventricular or arcuate nucleus. We found delicate VIP-expressing nerve fibers from the SCN most likely projecting directly to OXT neurons in the PVN as demonstrated in the rat (Buijs et al., 1999). These nerve fibers were located close to TH immunoreactive cells but only in close apposition to neurons that all coexpress OXT and the VPAC1 receptor. DA neurons in the arcuate nucleus and in the ME did not coexpress VPAC1-IR. Although there could be species differences between rats and mice, our observations in mice do not support a direct inhibitory role of VIP-targeting DA neurons involved in the circadian PRL secretion rhythm observed in the mating or cervical stimulated female rat. When DA antagonists are injected corresponding to the N and at D surge, the rise in PRL does not seem to be explained by the block of DA alone (Arey and Freeman, 1989). These findings indicate that besides the release of the tonic inhibition of DA, a releasing or stimulating factor is involved in circadian PRL secretion rhythm. This releasing factor could be OXT modulated by VIP *via* the VPAC1 receptor. Earlier studies have shown that injections of VIP antisense nucleotide into the SCN block circadian rhythmic release of both PRL and OXT in cervical stimulated ovariectomized rats (Egli et al., 2004), and injection of OXT and VIP antagonists both blocks the N and D surge of PRL induced by mating (Arey and Freeman, 1990). Another study found that blocking VIP only affects the N surge of PRL (Arey and Freeman, 1989, 1992b). The results in our study support a functional role of VIP as a modulator of OXT in the circadian release of PRL.VPAC1 expression was found not only on OXT neurons in PVN, but also on OXT neurons in SON, as well as in the small groups of neurons located on each side of the third ventricle in the basal hypothalamus. Whether these OXT neurons could be involved in the regulation of PRL needs further investigation. OXT is released from the posterior pituitary with a circadian secretion profile (Forsling, 2000). It is possible that VIP *via* the VPAC1 receptor could modulate OXT neurons linking VIP to the circadian releasing pattern of OXT from the posterior pituitary. However, this will need further investigation.

In our original article characterizing our VPAC1 receptor antibody (Fahrenkrug et al., 2000), a strong amplification system of biotinylated tyramide was used and showed VPAC1-IR in many blood vessels of the rat central nervous system (Fahrenkrug et al., 2000). In rats, the VPAC1 receptor was found to be present on the surface of smooth muscle cells of the cerebral blood vessels (Fahrenkrug et al., 2000), and this finding has later been reproduced by another group using another VPAC1 antibody (Erdling et al., 2013). VPAC1 receptor immunoreactivity has also been demonstrated in the porcine basilar artery (Grant et al., 2006). In mice, VPAC1-IR was found localized as in the rat, although this study focused on the expression of VPAC1 in neurons in the PVN and SON. VPAC1 neurons were visible by this technique, and we noticed a better signal/background using another amplification step of Envision© without losing the specific staining in the neurons. This was most likely due to a higher expression level of VPAC1 in neurons of the PVN and SON compared to that found in the cerebral blood vessels as supported by *in situ* hybridization of VPAC1 mRNA in the PVN. The findings highlight the importance of choosing the amplification system best designed for the assignment.

## Conclusion

VIP-expressing neurons from the ventral SCN project to the magnocellular neuroendocrine OXT neurons. The present study demonstrates that the OXT neurons in the mouse hypothalamus exclusively coexpress the VPAC1 but not the VPAC2 receptor. No VIP receptors were found on DA neurons in the periventricular or in the arcuate nucleus. The findings suggest that VIP signaling from the SCN does not directly target DA neurons involved in PRL secretion and support the notion that VIP from neurons in the SCN could regulate circadian release of OXT in the posterior pituitary and/or modulate OXT neurons as a releasing factor involved in the circadian regulation of PRL release from pituitary lactotrophs.

## Data Availability Statement

The raw data supporting the conclusions of this article will be made available by the authors, without undue reservation.

## Ethics Statement

The animal study was reviewed and approved by Danish Veterinary Authorities (Dyreforsoegstilsynet) license no. 2008/561-1445. The animal research ethics committee (Dyreforsoegstilsynet) granted a formal waiver of ethics approval license no. 2017-15-0201-01364 to JH and thereby approved the study.

## Author Contributions

IS and JH: conceptualization and formal analysis. JH: methods, imaging and figures, and writing—original draft. IS: wrote the first version. IS and JH: wrote the final version. All authors contributed to the article and approved the submitted version.

## Conflict of Interest

The authors declare that the research was conducted in the absence of any commercial or financial relationships that could be construed as a potential conflict of interest.

## Footnotes

^1^http://www.bitplane.com

^2^http://mouse.brain-map.org/gene/show/22112

## Supplementary Material

SUPPLEMENTARY FIGURE 1 Distribution of VPAC1 immunoreactivity in the mid part of PVN in a VPAC1 knockout mouse. The VPAC1 mice were originally characterized by Fabricius et al. (2011). These mice had exon 4–6 deletion making a functional knockout, but a truncated part of the N-terminal receptor protein was still expressed resulting in a positive staining using our VPAC1 antibody (see also “Materials and Methods” section). Scale bars: = 100 μm.

## Abbreviations

VIP: vasoactive intestinal polypeptide
PRL: prolactin
DA: dopamine
OXT: oxytocin
AVP: arginine vasopressin
TH: tyrosine hydroxylase
SCN: suprachiasmatic nucleus
PVN: paraventricular nucleus
SON: supraoptic nucleus.
