# Supplementary figures and images for: Localization of Vasoactive Intestinal Polypeptide Receptor 1 (VPAC1) in Hypothalamic Neuroendocrine Oxytocin Neurons; A Potential Role in Circadian Prolactin Secretion
Source: Front Neuroanat. 2020 Oct 29;14:579466. doi: 10.3389/fnana.2020.579466 (PMC7658414; doi:10.3389/fnana.2020.579466)

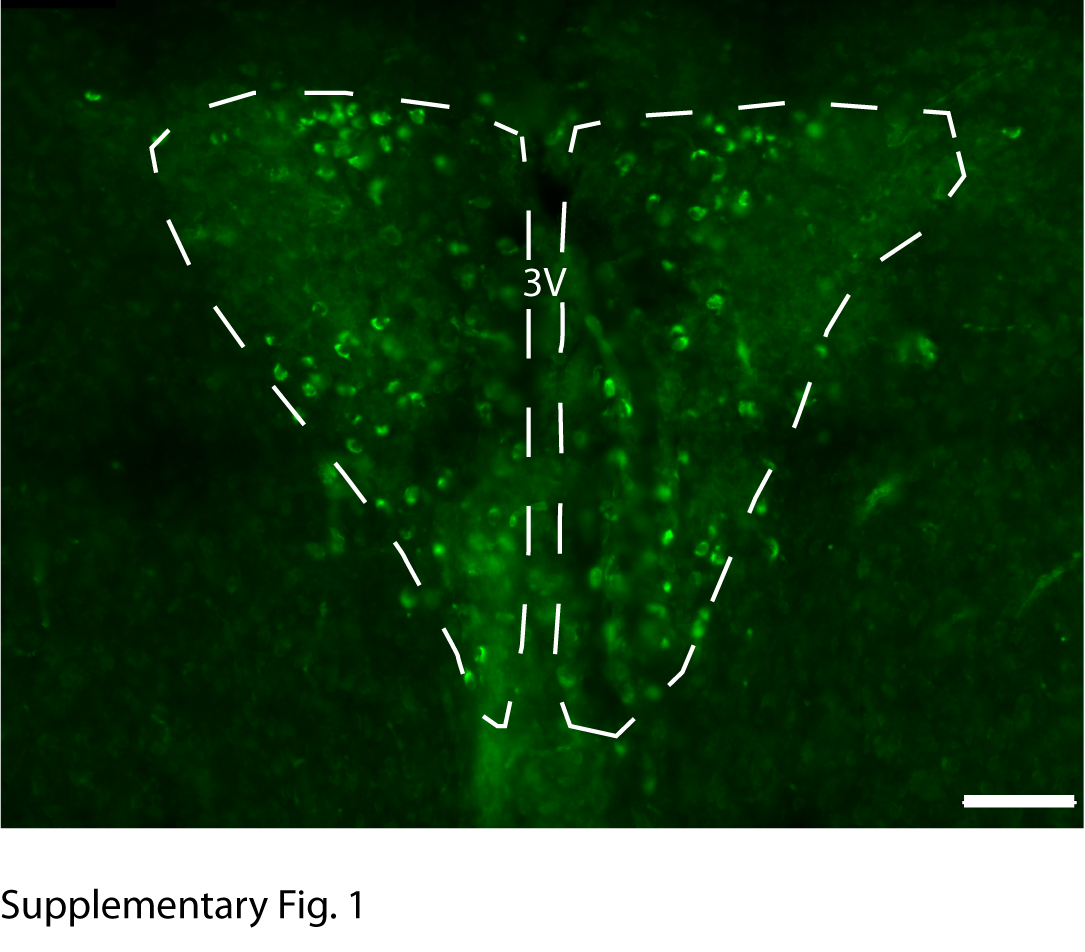

Supplement: SUPPLEMENTARY FIGURE 1 — Distribution of VPAC1 immunoreactivity in the mid part of PVN in a VPAC1 knockout mouse. The VPAC1 mice were originally characterized by Fabricius et al. (2011). These mice had exon 4–6 deletion making a functional knockout, but a truncated part of the N-terminal receptor protein was still expressed resulting in a positive staining using our VPAC1 antibody (see also “Materials and Methods” section). Scale bars: = 100 μm. [file Image_1.TIF]
